# Mapping Genetic Modifiers Affecting Photoreceptor Degeneration and Axial Length in Eyes from Homozygous *Mfrp^rd6^* Mice

**DOI:** 10.3390/ijms27209053

**Published:** 2026-10-13

**Authors:** Jai Pinkney, Sonia Weatherly, Gayle B. Collin, Belinda K. Cornes, Lisa Stone, Patsy M. Nishina, Jürgen K. Naggert

**Affiliations:** The Jackson Laboratory, Bar Harbor, ME 04609, USA; jai.pinkney@gmail.com (J.P.); soniawxly@gmail.com (S.W.); gayle.collin@jax.org (G.B.C.); belinda@doppeldata.au (B.K.C.); lisa.stone@jax.org (L.S.); patsy.nishina@jax.org (P.M.N.)

**Keywords:** *Mfrp*, modifier, axial length, photoreceptor degeneration, QTL analysis

## Abstract

MFRP (Membrane Frizzled-Related Protein) is a member of the frizzled-related family of proteins with currently unknown molecular function in the eye. Mutations in the *MFRP* gene in humans cause microphthalmia, nanophthalmia, retinitis pigmentosa, and foveoschisis, and are also associated with refractive errors. The clinical presentations among patients with MFRP mutations varies, with some showing one of the abnormalities and others combinations of disease phenotypes, suggesting the action of modifier genes. Unlike genetic studies in humans, localization and eventual identification of modifier genes in mouse models is straightforward. Here we report the chromosomal localization of five novel quantitative trait loci (QTL) affecting the degree of photoreceptor degeneration and axial length in a mouse model carrying the homozygous *Mfrp^rd^*^6^ allele. QTL analysis was performed on genotype and phenotype data from 225 F2 progeny homozygous for the *Mfrp^rd^*^6^ mutant allele derived from an intercross of (B6.Cg-*Mfrp^rd^*^6^*^/rd^*^6^ × C3A.BLiA-*Pde6b*^+^/J) F1 mice. Three QTL peaks were detected for number of photoreceptor nuclei, a measure of retinal degeneration, on Chr2 (53.3 centimorgan (cM)), Chr4 (56.6 cM), and Chr12 (45.5 cM). Two QTL were detected for axial length on Chr3 (49.5 cM) and Chr19 (24.5 cM). The results demonstrate that diverse *Mfrp*-associated disease phenotypes can be genetically modified, indicating that genetic variation underlies phenotypic variation.

## 1. Introduction

Membrane frizzled-related protein (MFRP) is a transmembrane protein with multiple domains: two cubilin (CUB), a low-density lipoprotein receptor a (LDLRA), and a cysteine-rich domain (CRD) [1]. It is expressed in the retinal pigment epithelium (RPE) and ciliary body epithelium [2,3] of the eye and localizes to the apical processes of the RPE [1]. Disruptions in MFRP have been associated with several human ocular diseases, including hyperopia [4], nanophthalmos 2 (NNO2, OMIM #609549) [5,6], posterior microphthalmia 5 (MCOP5, OMIM #611040) [7], retinitis pigmentosa [8], foveoschisis [9,10], a syndrome associated with optic disk drusen [10,11,12,13], and decreased vascular density [14]. Mouse models with mutations in *Mfrp*, including *rd6* (c.445+3_445+6delAAGT) [2], *rdx* (c.174delG) [15], and a c.498_499insC knock-in [16], develop disease phenotypes similar to those observed in human *MFRP*-associated diseases, namely hyperopia, reduced axial length (AL), retinal degeneration associated with decreased electrophysiological responses [1,17], and RPE atrophy [15]. The *Mfrp* mouse models also present with uniformly sized and panretinally distributed white spots [15,17], similar to the white flecks reported in patients with mutations in *MFRP* [12,13] and in the human flecked retinal disorder, retinitis punctata albescens [2,17,18].

The molecular function of MFRP is currently not known [19]. Biochemical studies have suggested that MFRP may participate in docosahexaenoic acid (DHA, 22:6) enrichment [19] and in lipid homeostasis in RPE and PR cells [20,21]. MFRP has also been shown to be necessary for the RPE-specific expression of *Adipor1* [22] and the correct localization in the RPE of ADIPOR1 and KCNJ13 [21], and it has been proposed to act as a hub in organizing the RPE apical membrane [21]. MFRP, based on protein sequence and structural similarities, is a member of the frizzled-related family of proteins, and as such has been proposed to play a role in Wingless related integration site (WNT) signaling [2,23]. Interaction with WNT ligands and activation of the WNT signaling pathway have, however, yet to be demonstrated. The known protein–protein interactions described for MFRP include C1QTNF5, its bicistronic partner [3]; pleiotrophin, a secreted growth factor [24]; as well as ADIPOR1 and KCNJ13 [21].

A powerful method to gain insight into pathways that are perturbed or activated by a primary disease-causing mutation is the identification of modifier genes [25,26,27]. Genetic modifiers are variants of genes that arise naturally or are induced, whose actions in the presence of a primary mutation lead to alterations of the disease phenotype that normally occurs as a result of a primary mutation. Such alterations can include an increase in disease severity, a suppression of the primary phenotype or the appearance of a totally different disease phenotype (e.g., pleiotropic effects). Modifiers, on their own, do not necessarily cause a disease phenotype and can act either in an epistatic or additive manner [28,29]. Identification of genetic modifier genes is a useful tool to identify biological pathways that lead from a mutation to an observed phenotype and can provide additional information about what processes the mutated protein participates in and what its biological function may be. For a primary mutation in *Mfrp*, loci affecting the number of nuclei in the outer nuclear layer (ONL) as a measure for photoreceptor degeneration have previously been located on chromosomes 1, 6 and 11 in a cross of B6.Cg-*Mfrp^rd^*^6^/J with CAST/EiJ [30]. Genetic interactions of *Mfrp* were also reported with *Adipor1* [31], as well as *Adamsts19* and *Prss56* [32].

Here we report the genetic mapping of additional variants that modify the extent of photoreceptor degeneration and AL induced by the *Mfrp^rd^*^6^ mutation, the first step in identifying modifier genes. Analyzing the segregation of marker loci in F2 animals, homozygous for the *Mfrp^rd^*^6^ mutation, from an intercross between B6.Cg-*Mfrp^rd^*^6^/J and C3A.BLiA-*Pde6b^+^*/J, we determined the location of three QTLs for photoreceptor degeneration and two QTLs for AL. We also identify potential candidate genes within the QTL genetic intervals.

## 2. Results

### 2.1. Photoreceptor Degeneration and AL in Eyes of a B6.Cg-Mfrp^rd6^/J × C3A.BLiA-Pde6b^+^/J F2 Population

One hundred and five male and one hundred and twenty female mice, for a total of 225 F2 mice from a (B6.Cg-*Mfrp^rd^*^6^/J *×* C3A.BLiA-*Pde6b^+^*/J) F1 intercross population homozygous for the *Mfrp^rd^*^6^ allele, were phenotyped for both ONL thickness, through assessing the number of photoreceptor nuclei across the width of the ONL, and for AL. At four months of age, homozygous animals presented with variable nuclei counts (Figure 1B) and AL (Figure 1C). Six measurements of photoreceptor nuclei in the ONL, 500 µm on either side of the optic nerve head were averaged and ranged from ~4 to 8 rows (Figure 1A,B), indicating variable photoreceptor degeneration compared to B6/J and homozygous *Mfrp^rd^*^6^ mice which averaged 10 to 12 rows and 5 to 6 rows, respectively. AL varied from 3.05 to 3.55 mm (Figure 1C).

ONL nuclei counts (Figure 1B) and axial lengths (Figure 1C) were normally distributed in the F2 population (D’Agostino–Pearson test, K2 = 2.398, *p* = 0.302 and Kolmogorov–Smirnov test, KS = 0.054, *p* = 0.1, respectively) and consequently the data were not transformed for the QTL analysis. A Student’s *t*-test was performed to assess the effect of sex among phenotypes. Rows of PR nuclei and AL were not significantly different between males and females (*p* = 0.50 and *p* = 0.11, respectively); thus, male and female cohorts were combined for analysis.

### 2.2. Quantitative Trait Locus (QTL) Mapping

DNA from the F2 animals was genotyped using 62 simple sequence repeat markers approximately evenly distributed across the genome (Supplemental Table S1). The genome scan data are reported in supplemental Table S2. The marker data were used to generate pseudo-markers at 1 cM intervals for each chromosome. Left and right eyes from 225 F2 mice were phenotyped and initially analyzed separately (Supplemental Figure S1). Phenotype data are presented in the supplement; photoreceptor degeneration data (Table S3) axial length data (Table S4), and C3H and Het control data (Table S5). 

To determine thresholds for QTL detection, 1000 permutations of the phenotype data were performed and thresholds were calculated at the 1, 5, and 10% levels. The analyses were performed using the QTL mapping program R/qtl2 [33].

#### 2.2.1. Retinal Degeneration

Initial analysis of the left and right eyes showed similar QTL plots (Supplemental Figure S1), therefore, the analysis was repeated with the combined data. The resulting QTL plot is shown in Figure 2.

Two significant QTL were detected for retinal degeneration at the 0.01 level, located on chromosomes 2 and 4 with logarithm of the odds (LOD) scores of 9.8 and 7.4, respectively (Table 1). An additional suggestive QTL was found on chromosome 12 with a LOD score of 3.4 (*p* = 0.05). QTL effects are shown as plots of raw phenotypes for each genotype group in Supplemental Figure S2.

#### 2.2.2. Axial Length Measurements

As with the degree of photoreceptor degeneration, AL of the eye also did not differ between left and right eyes (Supplemental Figure S1). In the combined analysis, one significant (*p* = 0.01) and one suggestive (*p* = 0.05) QTL peak on chromosomes 3 and 19, respectively, were detected (Figure 3 and Table 2). QTL effects are shown as phenotype plots for each genotype group in Supplemental Figure S2.

### 2.3. Candidate Genes

The confidence intervals for each of the QTL identified are quite large, varying in size from 26 (Chr19) to 96 (Chr2) megabases (Mb), containing between 262 (Chr.19) and 865 (Chr2) protein-coding genes (Table 3). In order to prioritize the search for causative variants within the QTL intervals, we examined the regions for potential candidate genes. We used the Mouse Genome Informatics (MGI) ‘Human-Mouse: Disease Connection’ query tool to search the intervals for genes associated with a “vision/eye phenotype.” We used this high-level search term to avoid missing any eye-related phenotypes. The returned gene records in MGI were individually examined for their specific phenotypes and their relevance to retinal degeneration or AL. Phenotypes such as retinal degeneration, abnormal retinal morphology, reduced retinal thickness, retinal development, microphthalmia, abnormal eye cup, refraction, and others were retained. This yielded lists of ~20 genes per QTL, which were then queried for single nucleotide polymorphisms (SNPs) in their coding regions or in their splicing signal sequences between B6/J and C3H/HeJ, the closest inbred strains to the parentals of the mapping cross (MGI, Search Mouse SNPs). The sequence variants obtained from the database were confirmed by Sanger sequencing of the parentals of our mapping cross. The final list of candidate modifier genes is presented in Table 3 and the variant genotypes and chromosomal positions in Table S6.

## 3. Discussion

It has been observed previously that genetic background affects the phenotype of mice carrying the mutant *Mfrp^rd^*^6^ allele, even under conditions where environmental and experimental variation is minimized [30]. *Mfrp* is part of a growing list of ocular disease genes in mice that vary in phenotypic severity depending on genetic background [34]. The existence of at least two ocular syndromes associated with human *MFRP* mutations [5,12,35] and phenotypic variation within families [35,36] may also reflect an influence of genetic background. In human studies, identifying the role of genetic background in MFRP-associated disease is problematic due to the small number of affected individuals in the human population. In contrast, the mouse has well established genetic and molecular tools and techniques, which are available to identify modifier genes.

Here we report on the first step in identifying modifier genes, determining the chromosomal location for genes that affect photoreceptor cell loss as well as AL in mice homozygous for the *Mfrp^rd^*^6^ mutation. Using an F2 intercross between B6.Cg-*Mfrp^rd^*^6^/J and C3A.BLiA-*Pde6b*^+^/J, and genotyping and phenotyping F2 offspring that were homozygous for the *Mfrp^rd^*^6^ mutation, allowed us to localize three loci affecting photoreceptor cell number as a measure for photoreceptor degeneration, and two loci for AL of the eye as a contributing factor to hyperopia. Although the suggestive loci on chr3 and chr12 reach genome-wide significance at the 0.05 level in the permutation test, the chr12 locus exceeded the threshold only modestly and its proportion of the variance explained (6.7%) is likely overestimated [37].

It has previously been suggested that MFRP participates in at least two separate molecular pathways leading to retinal degeneration and hyperopia, respectively [31,38]. The locations of the QTL for the two disease phenotypes in this study do not overlap. Although this result is not an indication of independent pathways, it is consistent with a two-pathway hypothesis. Also, the retinal degeneration QTLs identified in an F2 intercross done with CAST/EiJ [30] did not overlap with those identified in this study where the intercross partner is C3A.BLiA-*Pde6b*^+^/J, indicating strain specific modifiers that allow for a more wide-ranging identification of molecules and pathways that modify *Mfrp^rd^*^6^ disease phenotypes.

Due to the relatively small size of our mapping cross (225 homozygous animals), the QTL regions are large, containing between 262 and 865 genes. As an initial approach, we ascertained candidate genes by selecting genes from the QTL regions that have previously been associated with a relevant phenotype, either in spontaneous mutant or knockout models and in human eye diseases, using the MGI ‘Human-Mouse: Disease Connection’ query tool. For the *Mfrp/MFRP* photoreceptor degeneration phenotype, we used keywords such as retinal degeneration, retinal thickness, abnormal retina, etc. For AL, we used nanophthalmia, microphthalmia, eye development, etc. We then explored whether the selected genes carried coding sequence or splice site variants in C3H/HeJ compared to B6/J. For non-synonymous coding changes, we used MutPred2 [39] to assess potential pathogenicity of the amino acid change. This selection process yielded one to five candidate genes per QTL (Table 3). The identified candidate genes can now be tested by assessing their expression levels in homozygous C3A.BLiA-*Mfrp^rd^*^6^ vs. B6J-*Mfrp^rd^*^6^, crossing knock-out alleles to B6-*Mfrp^rd^*^6^ mice or by generating the modifier allele in B6-*Mfrp^rd^*^6^ mice via CRISPR homology-directed repair [40].

The disadvantage of the candidate gene approach is that uncharacterized genes, genes that had not been previously associated with eye diseases, or unreported polymorphisms would not be considered. An unbiased, conventional approach to identify the causative variant gene within a QTL interval, is to generate congenic lines that carry only the genomic region corresponding to one QTL from the modifier strain, in our case C3A.BLiA-*Pde6b*^+^/J, onto the B6-*Mfrp^rd^*^6^ genetic background, and carry out a fine mapping cross between the congenic line and B6-*Mfrp^rd^*^6^. Crossover events occurring within the QTL region can then be used to narrow the region where the gene responsible for the phenotype is located, until the region is small enough that all genes can be tested within the critical interval [41,42].

Although the QTL mapped in this study clearly modify the eye phenotypes induced by the *Mfrp^rd^*^6^ mutation, an open question remains—whether the QTL phenotypes depend on the presence of the *Mfrp^rd^*^6^ mutation or whether the genes underlying the QTL by themselves cause an eye phenotype and their effects are additive. Congenic lines carrying the individual QTL can be used to answer this question.

## 4. Materials and Methods

### 4.1. Animal Husbandry

Mice were bred and housed in the research animal care facility at The Jackson Laboratory (JAX) in individually ventilated boxes in a specific pathogen-free vivarium with a 12 h light/dark cycle. Animals were monitored daily and boxes changed weekly. Mice were fed a 6% fat mouse diet (LabDiet^®^ 5K52, St. Louis, MO, USA) ad libitum, and provided free access to acidified water. All mice were treated in accordance with the Animal Care and Use Committee at JAX (#AUS99089) and in compliance with the Association for Research in Vision and Ophthalmology (ARVO) statement for ethical care and use of animals.

### 4.2. Experimental Animals

F1 progeny from a B6.Cg-*Mfrp^rd^*^6^/J by C3A.BLiA-*Pde6b*^+^/J mating were intercrossed to produce F2 animals. Two hundred twenty-five F2 progeny homozygous for the *Mfrp^rd^*^6^ mutation were assessed for AL and degree of retinal degeneration through assessment of rows of nuclei in the ONL. To ascertain that a single *Mfrp^rd^*^6^ allele or genetic background alone had no effect on the disease phenotypes assessed, 37 F2 mice heterozygous for *Mfrp^rd^*^6^ and 41 F2 mice that were wildtype for the *Mfrp^rd^*^6^ gene were also phenotyped (Table S5).

### 4.3. Genotyping

#### 4.3.1. Mfrp^rd6^ Allele

F2 mice from the segregating intercross were genotyped for the *Mfrp^rd^*^6^ mutation, which is a 4 bp deletion in the splice donor sequence of *Mfrp* intron 4 [2]. The mice were genotyped by the Transgenic Genotyping Service of the Jackson Laboratory using the following primers, in the 5′ to 3′ direction: CACTACCACCCCAGCAAGGAC and CTTCTCCAGAGAGTGCCCTTG.

#### 4.3.2. Genome-Wide Scan

Sixty-two simple sequence-length polymorphic markers that differed between C57BL/6J and C3A.BLiA-*Pde6b*^+^/J, approximately evenly spaced across the genome, were selected for the genome scan. These markers and their locations are listed in supplemental Table S1.

### 4.4. PCR Amplification and Allele Typing

DNA was obtained by digesting mouse tail tips in 120 µL of 50 mM sodium hydroxide for 30 min at 90 °C, followed by neutralization with 30 µL of Tris (pH 8.0), and by centrifugation of the solution at 3600 RPM for 6 min. Each tube received a core mix of 11 μL, which consisted of 6.20 μL of dH_2_O, 2.00 μL of Cresol Red dye (Sigma Aldrich^®^, St. Louis, MO, USA), 1.10 μL of 10X Standard Taq Reaction Buffer (New England Biolabs^®^, Ipswich, MA, USA), 0.25 μL of 10 mM dNTP (New England Biolabs^®^), 0.20 μL of 10 mM of each primer, 0.05 μL of Taq DNA polymerase (New England Biolabs^®^), and 1.00 μL of DNA solution. The following cycling conditions were used: 94 °C, 2 min; [94 °C, 20 s; 50 °C, 30 s; 72 °C, 40 s] × 49; 72 °C, 7 min; 4 °C hold. The resulting PCR products were resolved by gel electrophoresis in a mixture of 3% Metaphor® and 1% SeaKem® LE agarose (Lonza Rockland, Rockland, ME, USA).

### 4.5. Assessment of AL

At four months of age, F2 animals were euthanized by carbon dioxide asphyxiation and cervical dislocation. Eyes were enucleated and placed in cold 1X PBS to prevent desiccation. The AL of eyes from each mouse was immediately measured 3 times with Vernier calipers and averaged, using the method described in Collin et al. [43]. Measurements were carried out blinded to the genotype of the animals.

### 4.6. Assessment of Retinal Degeneration

Eyes were fixed in cold methanol acetic acid 1× PBS (3:1:4) overnight. Paraffin embedding, sectioning, and staining of mounted sections with hematoxylin and eosin (H&E) was conducted following the procedure described in Won et al. [1]. Stained H&E sections of eyes were scanned on a Hamamatsu NanoZoomer (Hamamatsu City, Shizuoka Pref., Japan) to provide high-resolution digital data for quantification of remaining nuclei. The sections were viewed using NDP.view 2 (Hamamatsu). Rows of photoreceptor nuclei in the outer nuclear layer were counted to assess the degree of photoreceptor degeneration using this software. The software ruler was used to measure a distance 500 µm away from the optic nerve on each side of the eye. The number of nuclei were then counted in three non-adjacent columns oriented along the width of the ONL, for a total of 6 measurements. These measurements were averaged to obtain a count of remaining photoreceptor nuclei per column. Counts were carried out while blinded to the genotype of the animals.

### 4.7. QTL Analysis

QTL analysis of the genotype and phenotype data from the F2 cross was conducted using R/qtl2 [33]. The distributions of photoreceptor nuclei rows and AL were not statistically different from a normal distribution as assessed using the D’Agostino–Pearson and Kolmogorov–Smirnov tests in Prism 10.5 (GraphPad Software, Boston, MA, USA). Therefore, there was no requirement to transform the data. Any samples that were 3 standard deviations away from the mean of a particular phenotype were excluded from the analysis. Final sample size for the photoreceptor nuclei count was 225 and for the axial length measurement was 224. A recombination fraction plot of the marker genotype data was generated and the genotype quality was deemed acceptable. The F2 genotyping data were used to generate pseudo-markers at 1-cM spacing for each chromosome. For each of the phenotypes analyzed, one thousand permutations of the phenotype data were performed to determine the thresholds for QTL detection [44] and obtain genome-wide LOD significance thresholds of *p* < 0.01, *p* < 0.05, *p* < 0.10, respectively. QTLs with a LOD score above the 1% threshold were considered significant. The variance explained for each QTL was estimated from the LOD score using the formula h^2^ = 1 − 10^−2*LOD/n^ [45]. Confidence intervals were established using 95% Bayes credible intervals in R/qtl2 [45].

### 4.8. Selection of Candidate Genes

Genes within critical QTL intervals were obtained using the ‘Genes and Markers’ query form (https://www.informatics.jax.org/marker; accessed on 19 August 2026) in the Mouse Genome Informatics (MGI) database (Mouse Genome Informatics, The Jackson Laboratory, Bar Harbor, Maine, URL: http://www.informatics.jax.org; accessed on 1 September 2026). The genes were filtered for their association with an eye phenotype using the ‘Human-Mouse: Disease Connection’ query tool (https://www.informatics.jax.org/humanDisease.shtml; accessed on 19 August 2026) The genes so selected were then examined for the presence of non-synonymous coding and splice site variants between C57BL/6J and C3H/HeJ using the ‘Mouse SNP’ query in MGI (http://www.informatics.jax.org/snp; accessed on 19 August 2026). The phenotype records in the individual gene records in MGI were examined manually for their relevance to photoreceptor cell loss and AL (for example retinal degeneration or nanophthalmos were considered relevant).

The presence of the SNPs in the selected genes of mutant mice was confirmed by Sanger sequencing. Cycle sequencing of DNA samples was performed using ThermoFisher’s BigDye Terminator reaction kit Version 3.1. Purified reactions were run on an Applied Biosystems 3730xl sequencer (Applied Biosystems, Waltham, MA, USA).

MutPred2 [39] was used to assess potential pathogenicity of the amino acid change predicted by the non-synonymous coding variant. We accepted a low cutoff score of 0.2 to guard against false negatives.

## Figures and Tables

**Figure 1:**
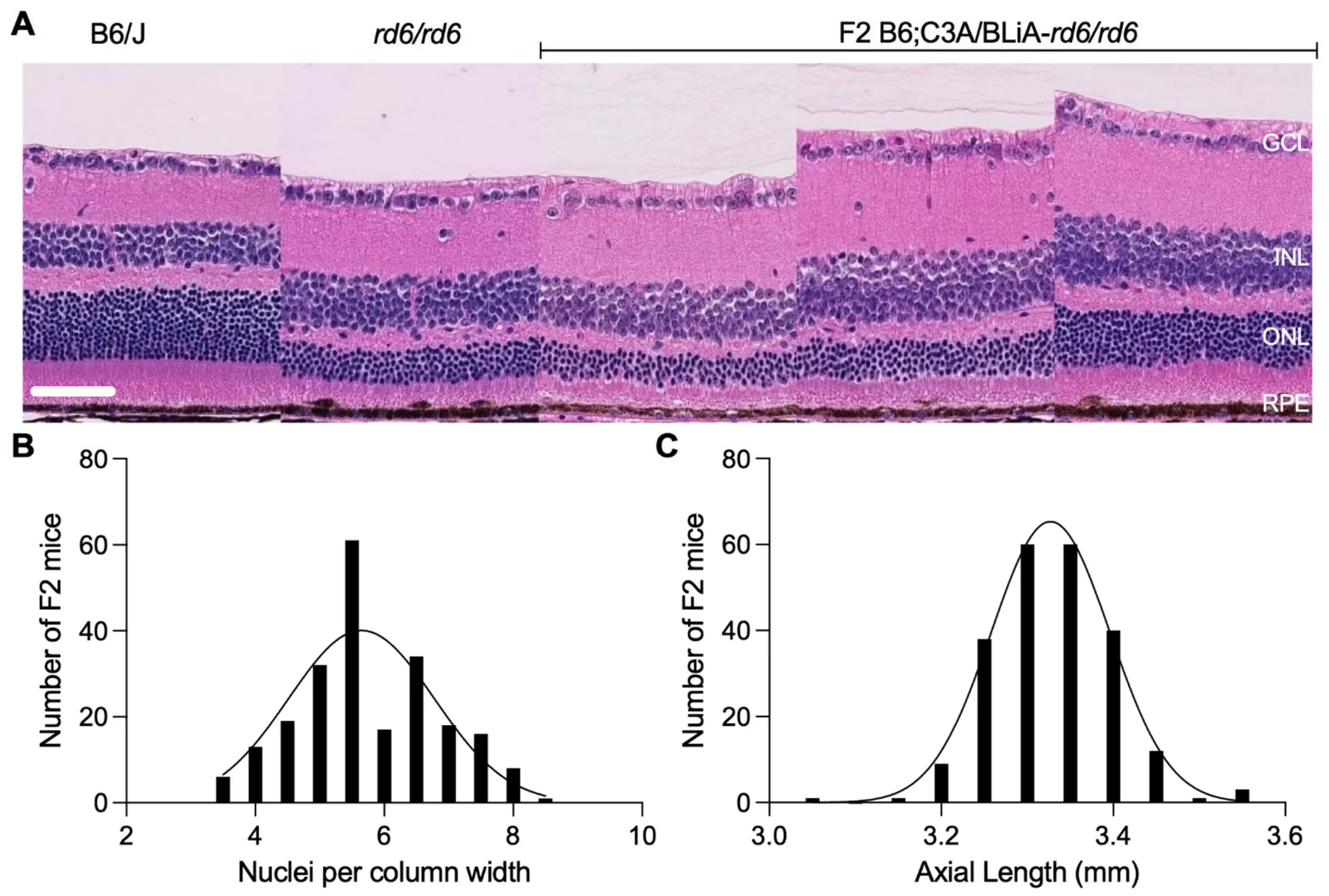
Representative cross sections of of B6/J (control) and homozygote *Mfrp^rd^*^6^ parentals as well as three F2 *rd6*/*rd6* retinas from the mapping cross stained with H&E (scale bar, 50 µm). Ganglion cell layer–GCL, inner nuclear layer–INL, outer nuclear layer–ONL, retinal pigment epithelium–RPE (**A**). Distribution of ONL nuclei in the mapping population measured in six vertical columns across the width of ONL for each section and averaged (**B**). Distribution of axial lengths of eyes in the mapping population (**C**).

**Figure 2:**
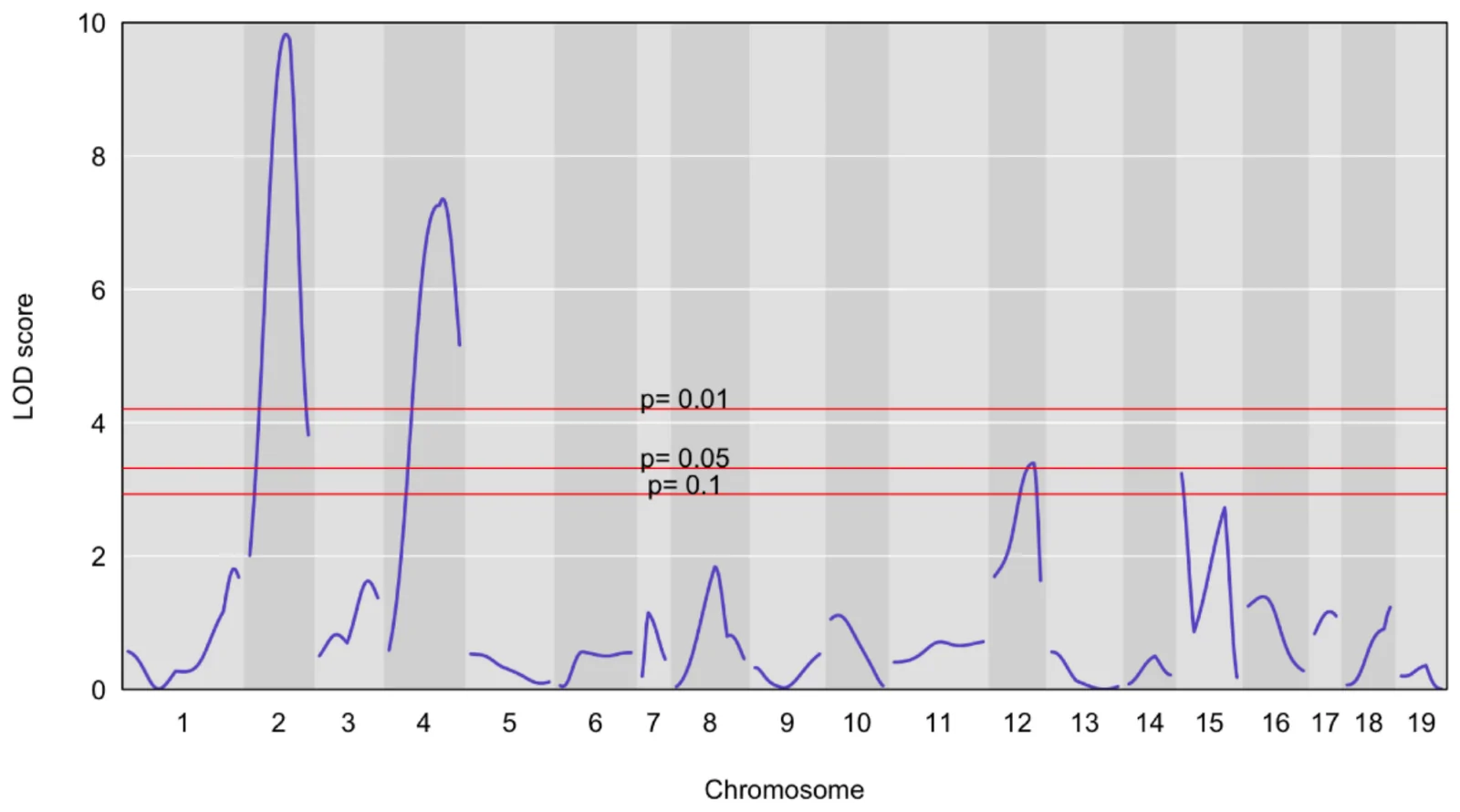
LOD score plot for rows of ONL nuclei across the width of the ONL. The peaks on chromosomes 2 and 4 represent genomic areas that are significantly associated with reduced rows of ONL nuclei.

**Figure 3:**
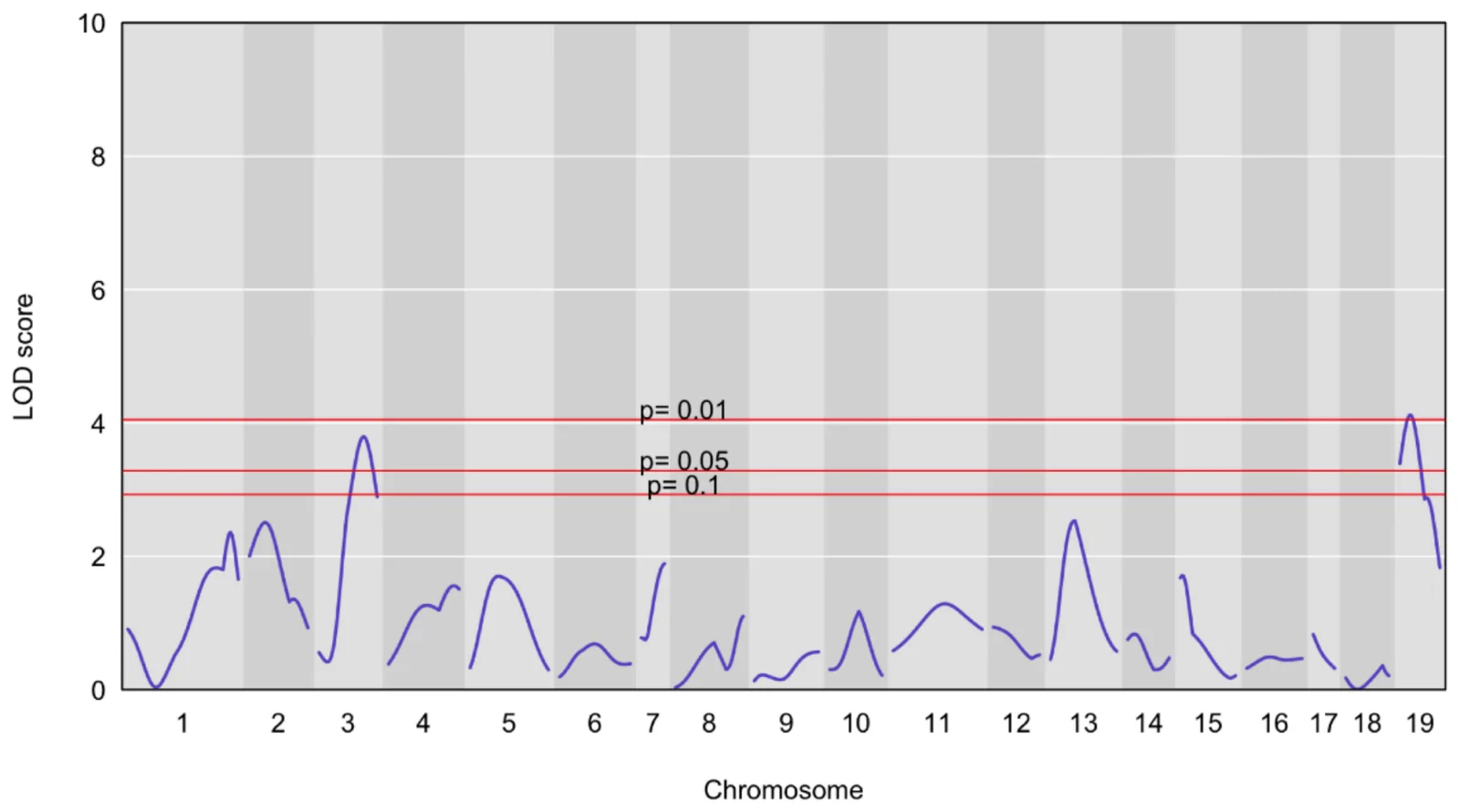
LOD score plot for AL. The peaks on chromosomes 3 and 19 represent genomic areas that are suggestively (*p* < 0.05) and significantly (*p* < 0.01) associated with reduced AL.

**Table 1:** Significant and suggestive QTL for retinal degeneration.

chr	pos [cM]	LOD	Variance Explained R^2^ [%]	ci_lo [cM]	ci_hi [cM]
2	53.3	9.8	18.2	27.3	68.9
4	56.6	7.4	14.1	18.6	68.9
12	45.5	3.4	6.7	17.5	50.3

Chromosome (chr), position (pos), LOD score (LOD), variance explained, and confidence interval (ci)—lower (ci_lo) and upper (ci_hi)— are listed.

**Table 2:** Significant and suggestive QTL for AL.

chr	pos [cM]	LOD	Variance Explained R^2^ [%]	ci_lo [cM]	ci_hi [cM]
3	49.5	3.8	7.5	17.5	59.0
19	24.5	4.1	8.1	17.5	45.9

Chromosome (chr), position (pos), LOD score (LOD), variance explained, and confidence interval (ci)—lower (ci_lo) and upper (ci_hi)—are listed.

**Table 3:** Candidate modifier genes for photoreceptor degeneration (Deg) and axial length (AL).

Class	chr	ci [cM]	#Genes	Eye	rel	Gene	Phenotype	Variant	MutPred2
Deg	2	27.3–68.9	865	180	24	*Cerkl*	RP26	C301R	0.512
						*Dll4*	human macula GWAS	Q419L, G627S	0.533
						*Galk2*	abnormal retinal morphology	P117R	0.476
						*Sirpa*	Abnormal ONL	N224H	0.347
						*Spg11*	RD, macular degeneration	T227M	0.437
	4	18.6–68.9	829	177	21	*Col9a2*	Peripheral retinal thinning	R610H	0.471
	12	17.5–53.0	370	98	22	*Tshr*	abnormal ERG	E382D	0.206
AL	3	17.5–59.0	722	191	28	*Dclre1b*	aphakia, abnormal optic cup	P184H, N195I	0.75, 0.633
						*Cgn*	Epithelial proliferation	Q638R	0.085
	19	17.5–45.9	262	73	31	*Cep55*	abnormal cytokinesis,	G55D	0.323
						*Hps1*	anterior retinal morphology	Y89S	0.745
						*Pdzd7*	Rod-cone dystrophy	D525E	0.132

Class: Deg—Degeneration; AL—axial length; chr—chromosome; ci—confidence interval; #genes—within the ci; eye—#of genes associated with vision/eye; rel—# of genes with a phenotype relevant to retinal degeneration or AL; phenotype—mutant phenotype associated with the gene; (RD, retinal degeneration) variant—protein polymorphism between B6/J and C3H/HeJ; MutPred2 score (higher values are considered more pathogenic).

## Data Availability

Data are contained within the article or supplementary material.

## Supplementary Materials

The following supporting information can be downloaded at: https://www.mdpi.com/article/10.3390/ijms27209053/s1.
