# Supplementary material for: Mapping Genetic Modifiers Affecting Photoreceptor Degeneration and Axial Length in Eyes from Homozygous Mfrprd6 Mice
Source: Int J Mol Sci. 2026 Oct 13;27(20):9053. doi: 10.3390/ijms27209053 (PMC13705533; doi:10.3390/ijms27209053)
Supplement: Supplementary file 1 [file ijms-27-09053-s001.zip › ijms-4569977-supplementary captions.pdf]

Figure S1: Covariates and LOD score plots for left and right eyes;
Figure S2: QTL effects. Plots of raw phenotypes for each genotype group;
Table S1: MIT Simple Sequence Repeat markers used;
Table S2: Genome scan data;
Table S3: Photoreceptor degeneration data;
Table S4: Axial length data;
Table S5: C3H and Het control data;
Table S6: Variant genotype and position.
