# Supplementary material for: Mapping Genetic Modifiers Affecting Photoreceptor Degeneration and Axial Length in Eyes from Homozygous Mfrprd6 Mice
Source: Int J Mol Sci. 2026 Oct 13;27(20):9053. doi: 10.3390/ijms27209053 (PMC13705533; doi:10.3390/ijms27209053)
Supplement: Supplementary file 1 [file ijms-27-09053-s001.zip › Supplemental Figure S1.pdf]

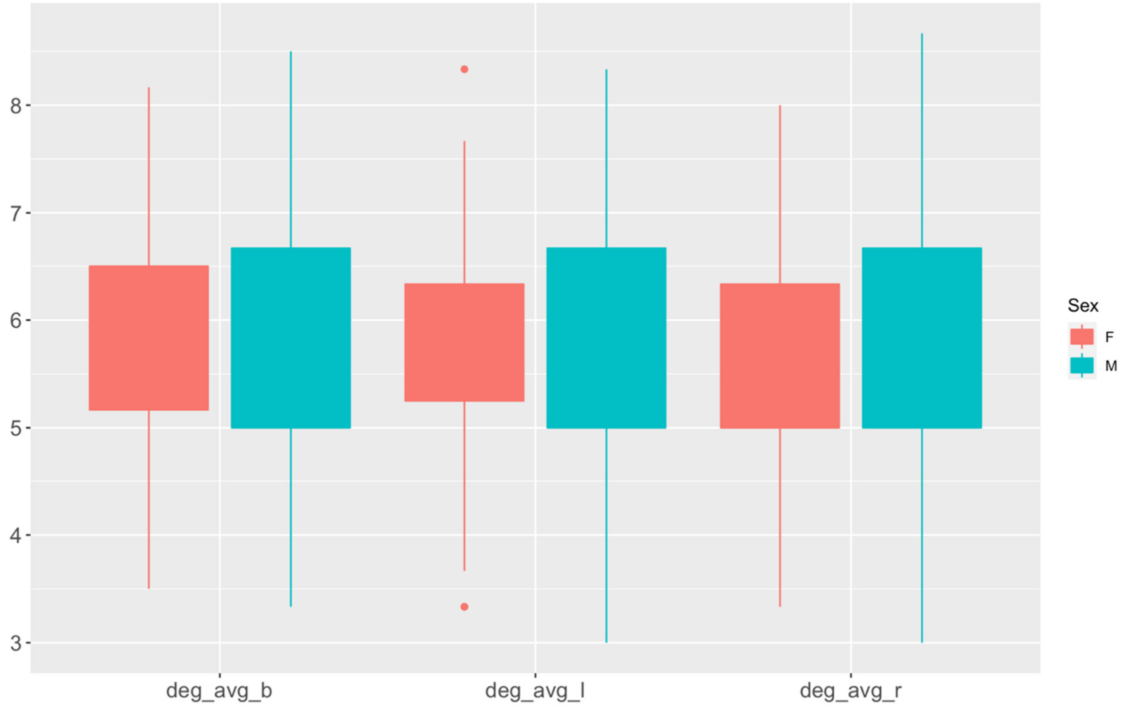

A: Photoreceptor Nuclei counts for both, left, and right eye by sex. There were no significant sex or laterality differences.

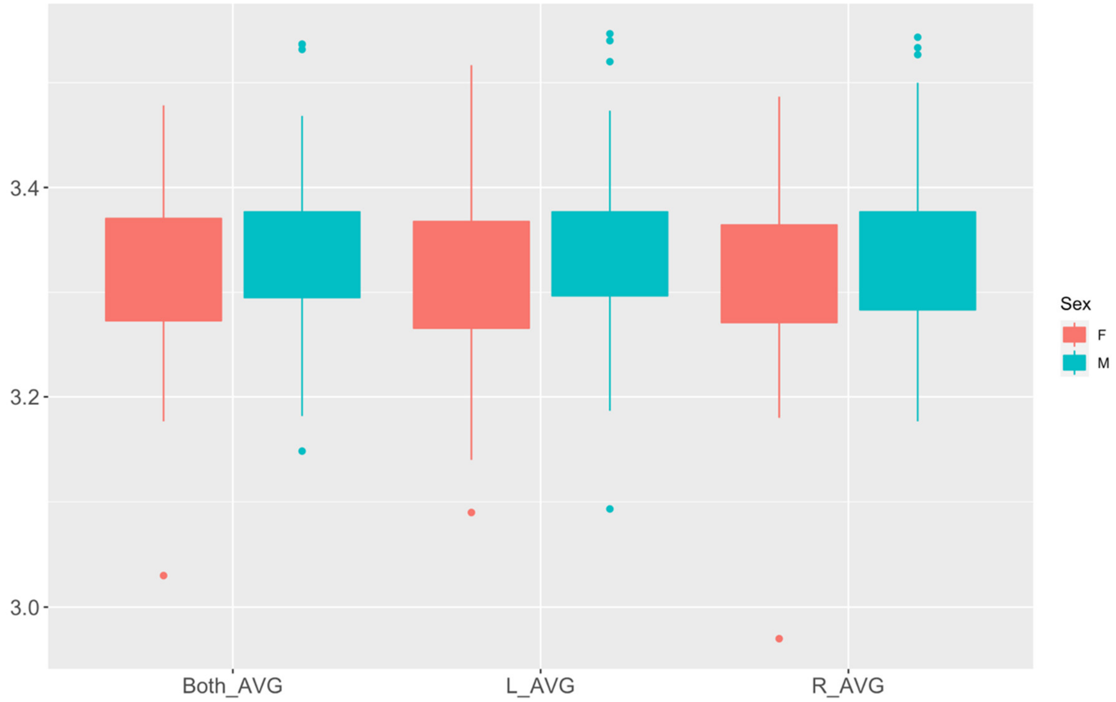

B: Axial length measurements for both, left, and right eye by sex. There were no significant sex or laterality differences.

A two-sample t-test was performed to assess the effect of sex among phenotypes. The test measures whether the average (expected) phenotypic value differs significantly between female and male groups.

H0: The average of each phenotype for female and male may be assumed to be equal.

Table A: Group means and p-values

phenotype	p.value	female_mean	male_mean
**R_AVG**	0.1101536	3.319076	3.333622
**L_AVG**	0.1142482	3.326849	3.341779
**Both_AVG**	0.1127507	3.323074	3.337470
**deg_avg_r**	0.4551169	5.711111	5.825397
**deg_avg_l**	0.5970057	5.730556	5.809524
**deg_avg_b**	0.4971512	5.720833	5.820635

1. We observe a large p-value (p-value > 0.05) for all phenotypes (after the removal of outliers), so we cannot reject the null hypothesis of identical average scores.
2. No p-value was smaller than 0.05, so the null hypothesis of equal averages was not rejected for any trait.
3. Therefore the sex covariate was not added to the analysis

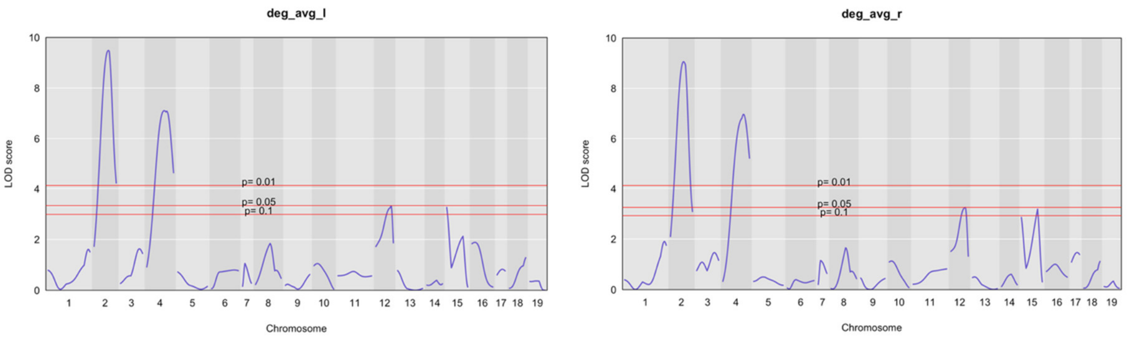

C: LOD score plots for photoreceptor nuclei count of left and right eye.

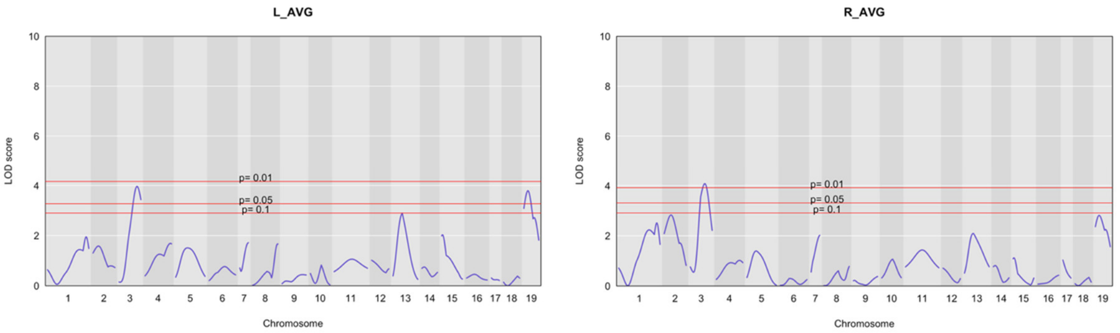

D: LOD score plots for axial length measurements of left and right eye.
