# Supplementary material for: Mapping Genetic Modifiers Affecting Photoreceptor Degeneration and Axial Length in Eyes from Homozygous Mfrprd6 Mice
Source: Int J Mol Sci. 2026 Oct 13;27(20):9053. doi: 10.3390/ijms27209053 (PMC13705533; doi:10.3390/ijms27209053)
Supplement: Supplementary file 1 [file ijms-27-09053-s001.zip › Supplemental Figure S2.pdf]

Raw phenotype values for each genotype group

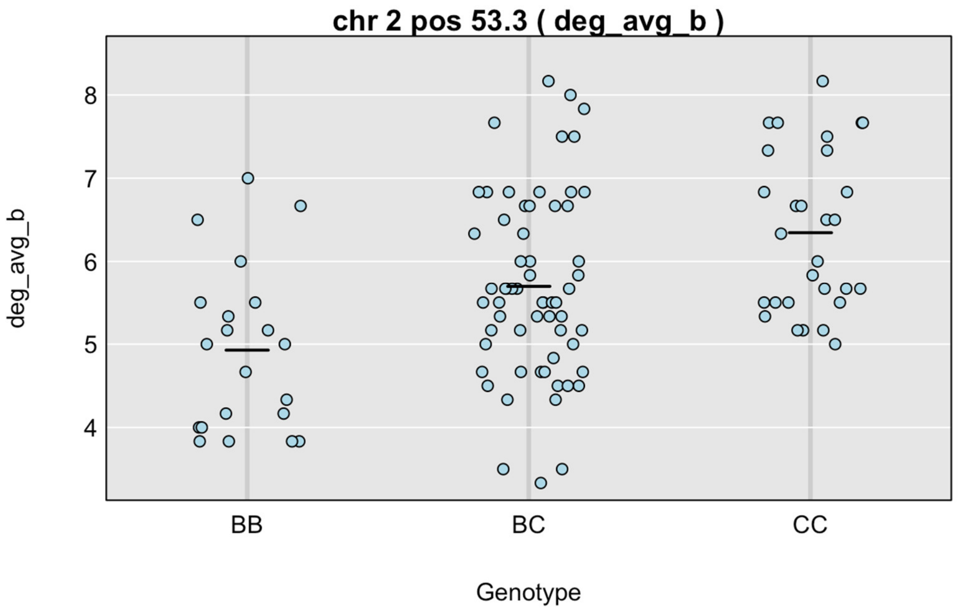

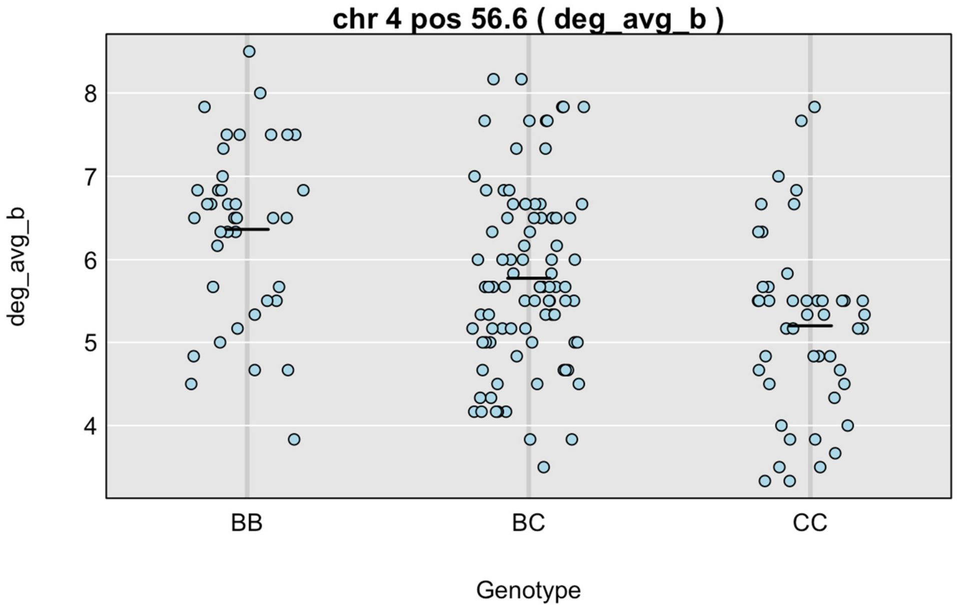

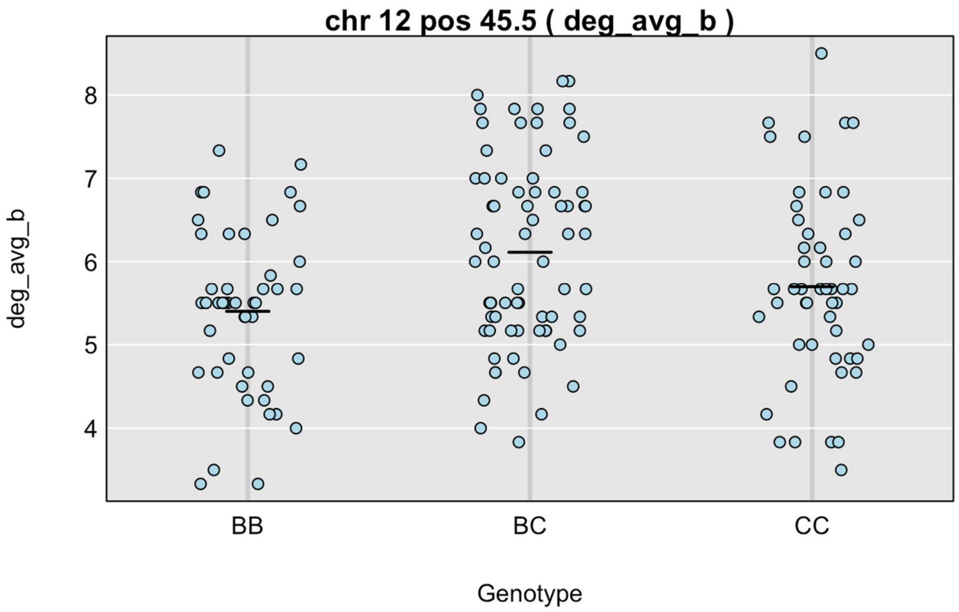
chr 12 pos 45.5 (deg_avg_b)
deg_avg_b
8
7
6
5
4
BB
BC
CC
Genotype

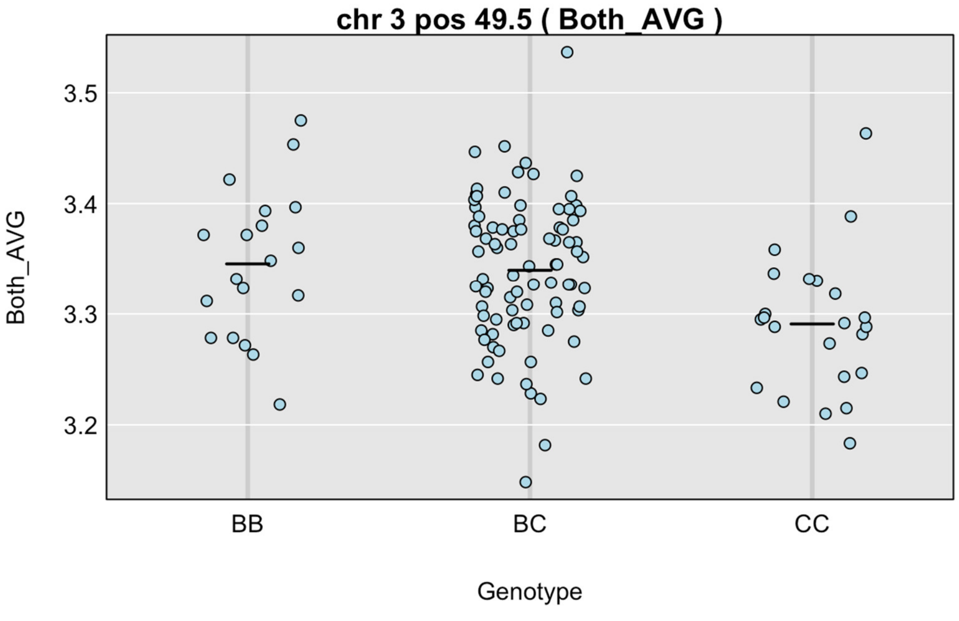
chr 3 pos 49.5 (Both_AVG)
Both_AVG
3.5
3.4
3.3
3.2
BB
BC
CC
Genotype

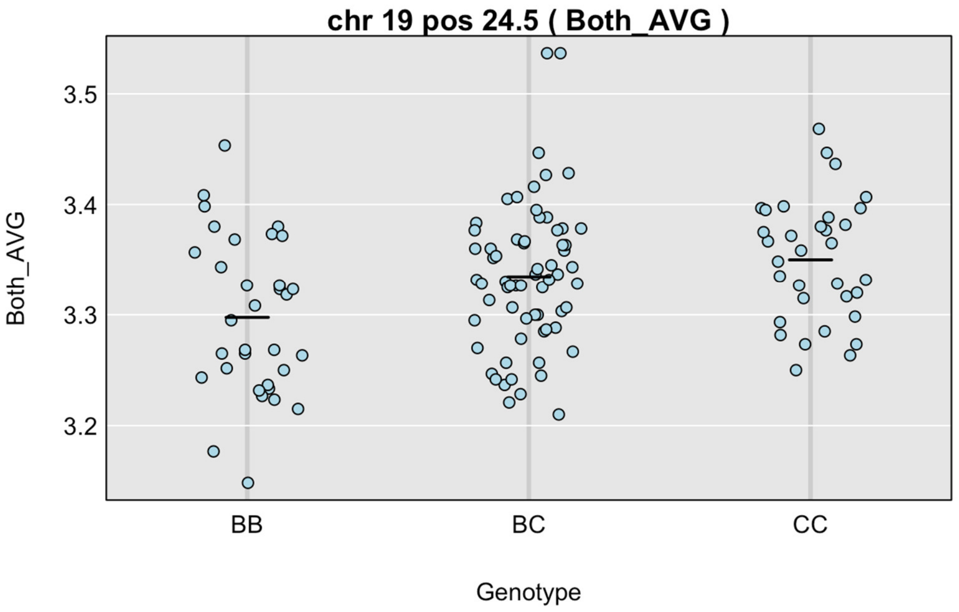

chr 19 pos 24.5 (Both_AVG)
3.5
3.4
3.3
3.2
Both_AVG
BB
BC
CC
Genotype
