# Supplementary material for: Mapping Genetic Modifiers Affecting Photoreceptor Degeneration and Axial Length in Eyes from Homozygous Mfrprd6 Mice
Source: Int J Mol Sci. 2026 Oct 13;27(20):9053. doi: 10.3390/ijms27209053 (PMC13705533; doi:10.3390/ijms27209053)
Supplement: Supplementary file 1 [file ijms-27-09053-s001.zip › Supplemental Table S1.pdf]

Supplemental Table S1 MIT Simple Sequence Repeat markers used.

MIT Marker	Position (cM)	B6	C3H	Chromosomal Location (m38)
1-3a	7.7	159	199	Chr1:19811970-19812128 bp
1-7	41.5	105	122	Chr1:74952818-74952921 bp
1-200	75.4	205	297	Chr1:150317922-150318120 bp
1-15	86.3	160	183	Chr1:168358674-168358833 bp
2-237	27.3	130	111	Chr2:40995481-40995603 bp
2-484	55.7	112	124	Chr2:118573430-118573541 bp
2-282	68.9	144	130	Chr2:148858318-148858457 bp
2-51	79.8	128	142	Chr2:163150442-163150568 bp
3-6	17.5	147	136	Chr3:48883405-48883548 bp
3-11	37.2	145	163	Chr3:100556675-100556821 bp
3-293a	59.0	146	134	Chr3:149269724-149269826 bp
4-286	18.6	96	76	Chr4:43196900-43196995 bp
4-12	54.6	198	168	Chr4:124371164-124371360 bp
4-54	68.9	150	174	Chr4:137890537-137890684 bp
5-106	17.5	163	153	Chr5:44991378-44991539 bp
5-157	39.3	123	111	Chr5:100629942-100630065 bp
5-292	73.2	114	128	Chr5:140687038-140687148 bp
6-384	15.3	126	148	Chr6:55199866-55199990 bp
6-101	31.7	151	163	Chr6:94859651-94859795 bp

6-201	65.6	148	106	Chr6:146633481-146633626 bp
7-91	27.3	144	134	Chr7:58499072-58499217 bp
7-31	31.7	246	226	Chr7:87493929-87494171 bp
7-66	43.7	164	148	Chr7:119885996-119886157 bp
8-94	10.9	156	130	Chr8:31341658-31341811 bp
8-162	38.3	149	131	Chr8:78427884-78428030 bp
8-242	47.0	166	196	Chr8:101759576-101759741 bp
8-271	59.0	96	120	Chr8:114360122-114360217 bp
9-256	21.9	122	100	Chr9:48443938-48444055 bp
9-73	39.3	130	138	Chr9:71574345-71574472 bp
9-82	67.8	148	156	Chr9:119496744-119496894 bp
10-194	20.8	70	84	Chr10:46844307-46844387 bp
10-42	41.5	184	196	Chr10:82655104-82655287 bp
10-134	57.9	101	93	Chr10:104611721-104611820 bp
11-226	2.2	142	126	Chr11:8904955-8905093 bp
11-38	44.8	76	112	Chr11:87800754-87800864 bp
11-258	65.6	130	170	Chr11:107725518-107725646 bp
12-112	17.5	154	136	Chr12:46754801-46754950 bp
12-101	44.8	170	118	Chr12:102494840-102495006 bp
12-79	50.3	154	132	Chr12:109969663-109969809 bp
13-64	15.3	102	116	Chr13:45056966-45057068 bp

13-159	32.8	142	160	Chr13:92183421-92183558 bp
13-76	62.4	106	88	Chr13:110563270-110563377 bp
14-207	7.7	128	104	Chr14:22093405-22093528 bp
14-121	26.2	149	127	Chr14:48626981-48627130 bp
14-102	37.2	124	142	Chr14:65866444-65866566 bp
14-166	62.3	144	128	Chr14:105322173-105322314 bp
15-209	26.2	127	106	Chr15:61681464-61681588 bp
15-71	35.0	118	132	Chr15:79590436-79590561 bp
15-245	56.8	119	101	Chr15:94278551-94278669 bp
15-161	65.6	102	128	Chr15:97011059-97011157 bp
16-131	6.6	144	180	Chr16:7319042-7319181 bp
16-212	25.1	200	178	Chr16:34883923-34884123 bp
16-153	45.9	194	210	Chr16:87583364-87583632 bp
17-10	19.7	157	148	Chr17:47535095-47535252 bp
17-139	25.1	136	164	Chr17:52659266-52659396 bp
17-39	40.4	110	92	Chr17:74282094-74282202 bp
18-64	0.0	152	172	Chr18:6108404-6108555 bp
18-184	26.2	172	127	Chr18:66819243-66819414 bp
18-186	30.6	126	108	Chr18:72020418-72020542 bp
19-80	17.5	124	134	chr19:23,313,374-23,313,498
19-91	35.0	112	90	chr19:47,909,441-47,909,549
19-33	45.9	252	261	chr19:56,195,632-56,195,893
