# Supplementary material for: Mapping Genetic Modifiers Affecting Photoreceptor Degeneration and Axial Length in Eyes from Homozygous Mfrprd6 Mice
Source: Int J Mol Sci. 2026 Oct 13;27(20):9053. doi: 10.3390/ijms27209053 (PMC13705533; doi:10.3390/ijms27209053)
Supplement: Supplementary file 1 [file ijms-27-09053-s001.zip › Supplemental Table S6.pdf]

Table S6. Chromosomal location of candidate genetic variants

gene	chromosomal location Mb	Genomic variant B6/C3H	Protein variant
Cerkl	chr2:79342427	A/G	C301R
Dll4	chr2:119332155		Q419L
Dll4	chr2:119332778	G/A	G627S
Galk2	chr2:125738590	C/G	P117R
Sirpa	chr2:129457597	A/C	N224H
Spg11	chr2:121942365	G/A	T227M
Col9a2	chr4:120911489	G/A	R610H
Tshr	chr12:91504209	G/C	E382D
Dclre1b	chr3:103714697	G/A	P184H
Dclre1b	chr3:103714664	T/A	N195I
Cgn	chr3:94677936	T/C	Q638R
Cep55	chr19:38057964	G/A	G55D
Hps1	chr19:42759325	T/C	Y89S
Pdzd7	chr19:45021625	G/T	D525E

genome position of variant based on Mouse GRCm39/mm39 assembly. Accessed through University of California Santa Cruz Genome Institute, 9/2026 (https://genome.ucsc.edu/index.html)
